# Mechanism and clinical application of ozone therapy in treating skin ulcer diseases

**DOI:** 10.4103/mgr.MEDGASRES-D-25-00079

**Published:** 2026-04-11

**Authors:** Yizhi Pan, Baoxiang Wang, Xuyuan Kuang

**Affiliations:** 1 Department of Dermatology, The Third Xiangya Hospital of Central South University, Changsha, Hunan Province, China; 2 Health Management Center, Xiangya Hospital, Central South University, Changsha, Hunan Province, China; 3 Department of Hyperbaric Oxygen, Xiangya Hospital, Central South University, Changsha, Hunan Province, China

**Keywords:** angiogenesis, antimicrobial, diabetic foot ulcers, ozone gas bath, ozone hydrogel, ozone therapy, pain modulation, re-epithelialization, skin ulcer disease, topical oxygen therapy

## Abstract

**Facts**

1. Ozone therapy is emerging as a novel treatment for skin ulcers.
2. The mechanisms of ozone therapy include improved tissue oxygenation, oxidation, antimicrobial effects, enhanced circulation, immune modulation, and accelerated tissue repair.
3. Research has focused on the relationship between the ozone concentration and therapeutic efficacy, highlighting the importance of an appropriate concentration.

**Open Questions**

1. What are the optimal ozone concentrations and application methods for different types of skin ulcers?
2. What are the long-term efficacy and safety profiles of ozone therapy for skin ulcers compared with those of conventional treatments?
3. Which specific types of skin ulcers respond best to ozone therapy?

Ozone therapy not only has antibacterial effects but also has a series of biological effects. It can stimulate the production of antioxidant enzymes, increase the elasticity of red blood cell membranes, improve blood flow and tissue oxygenation, promote the release of growth factors, and activate cell metabolism and immune function. Through multi-target regulation, ozone therapy has shown significant efficacy in treating skin ulcer diseases such as diabetic foot ulcers and burns, with overall controllable safety. Currently, most research on ozone therapy focuses on the correlation between the ozone concentration and therapeutic efficacy. This review provides a systematic review of the mechanism and clinical research progress of ozone therapy in treating skin ulcer diseases. While ozone therapy has demonstrated notable effectiveness in treating diabetic foot ulcers and other conditions, more rigorous clinical research is necessary to refine treatment plans and create standard guidelines for its use. In the future, the integration of ozone therapy with precision medicine and innovative delivery methods could broaden its application in treating skin ulcer diseases.

## Introduction

Skin ulcer disease is a type of disease characterized by ulcers (open wounds) on the skin or mucous membranes, with a long course of disease and difficulty in wound healing. Its pathogenesis and pathological process are complex and involve abnormal inflammatory reactions, tissue necrosis, vascular damage, infection, etc.^1^,^2^ The difficulty in treating these wounds lies in their persistent inflammatory response, difficulty in recovery after tissue damage, poor blood circulation leading to ischemia, bacterial infections, and biofilm formation.

Ozone is a substance with strong oxidizing ability that has gradually gained attention in the medical field since the late 19^th^ century. In recent years, the potential of ozone in the treatment of skin diseases has once again sparked a research boom. Owing to the broad-spectrum antibacterial properties, significant anti-inflammatory effects and ability to promote tissue repair, ozone therapy is particularly suitable for the treatment of chronic ulcer diseases, such as diabetic foot ulcers, leg ulcers caused by varicose veins, and refractory infected wounds.^3^ Existing evidence has indicated that ozone can effectively suppress excessive inflammatory responses by regulating key signaling pathways, such as nuclear factor kappa-B and Toll-like receptor 2, and improve the progression of the disease by affecting the Th1/Th17 immune balance.^4^,^5^

To explore its therapeutic potential, we comprehensively summarized the mechanism, clinical application, and safety of ozone therapy in the treatment of skin ulcer diseases. Moreover, we analyzed the limitations and controversies of ozone therapy.

## Literature Retrieval Strategy

This review conducted a systematic literature search of multiple electronic databases, including PubMed, Web of Science, Springer Nature Link, Taylor and Francis, Wiley Online, and ScienceDirect, from the establishment of each database until June 2025. The retrieval strategy combines medical keywords (MeSH) and free keywords via Boolean logical operators (and/or), covering two conceptual domains: (1) intervention measures (“oxygen-ozone therapy,” “ozone therapy,” “ozone oil,” “ozone water,” “ozone self-blood therapy,” “ozone gas bath”), and (2) disease (“skin ulcer” or “skin ulcer” or “diabetes foot ulcer” or “venous leg ulcer” or “pressure ulcer” or “burn wound” or “refractory wound”).

The inclusion criteria included the following: (1) research on ozone therapy for skin ulcer diseases; (2) literature types: randomized controlled trials, observational studies, systematic reviews, meta-analyses, and basic mechanism studies (*in vitro*/*in vivo* studies); (3) the use of humans as research subjects or relevant animal models; and (4) English-language literature. The exclusion criteria were as follows: (1) no full-text abstracts available and (2) research specifically focusing on atmospheric ozone exposure.

Two authors (YP and BW) conducted title/abstract screening and full-text evaluation. Disagreements can be resolved through the third independent investigator (XK).

## Pathological Characteristics of Skin Ulcerative Diseases

### Abnormal inflammatory response

The ulcer areas continue to infiltrate neutrophils, macrophages, and proinflammatory factors, such as tumor necrosis factor-α, interleukin (IL)-1β, and IL-6. This persistent state of inflammation disrupts the normal balance between inflammation and repair.^6^ Excessive production of reactive oxygen species in the ulcer area damages the structure of cell membranes and DNA and inhibits the proliferation of fibroblasts and the synthesis of collagen.^7^ In addition, the activity of matrix metalloproteinases (such as matrix metalloproteinase-2 and matrix metalloproteinase-9) present in the ulcer area leads to degradation of the extracellular matrix and thus hinders the formation of granulation tissue.^8^ In addition, other mechanisms are also deeply involved in the abnormal regulation of inflammation. These mechanisms include dysfunction in the process of inflammation resolution; abnormal activation of pathways, such as the NOD-like receptor pyrin domain containing 3 inflammasome;^9^ and obstruction of M2 polarization of macrophages toward promoting repair.^10^ These factors together constitute an unbreakable chronic inflammatory microenvironment, which is the core reason why skin ulcers are difficult to heal.

### Tissue necrosis

The surfaces of ulcers are often covered with yellowish-black gangrene or necrotic tissue, which hinders the penetration of oxygen and the migration of cells. Local ischemia and hypoxia lead to excessive activation of autophagy or abnormal apoptosis of cells, further exacerbating tissue damage.^11^

### Vascular damage and ischemia

Endothelial damage triggers thrombosis and occlusion of microvessels, such as the common thickening of the vascular basement membrane in diabetic foot ulcers.^12^ The expression of proangiogenic factors such as vascular endothelial growth factor and fibroblast growth factor is insufficient, leading to structural disorders and increased leakage of new blood vessels.^13^

### Infection and biofilm formation

Bacteria such as *Staphylococcus aureus and Pseudomonas aeruginosa* form biofilms on the surface of ulcers, which can resist clearance by the immune system and the action of antibiotics.^14^ Continuous infection leads to excessive activation of neutrophils, which release more proteases and reactive oxygen species, forming a vicious cycle and further aggravating the condition.

**Figure 1:**
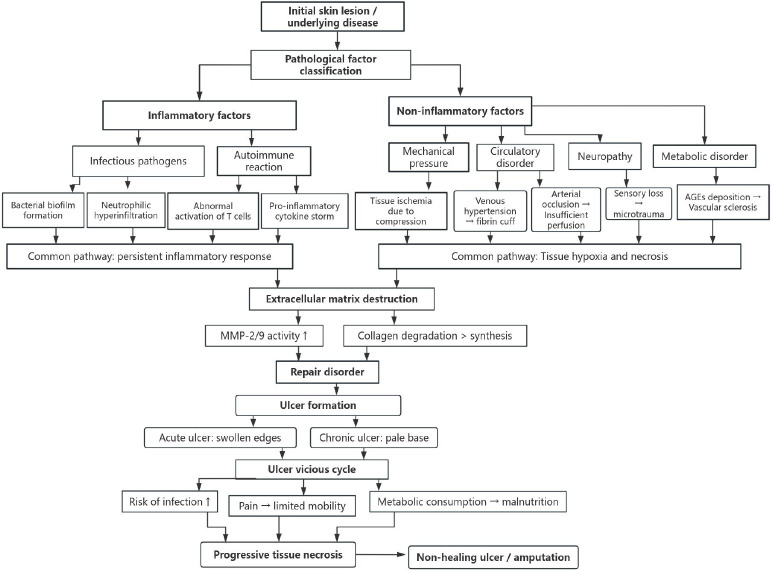
Pathological process of skin ulcerative diseases. Initial insults (e.g., mechanical pressure, circulatory disorders, infection, metabolic diseases) trigger distinct yet converging pathways dominated by inflammatory responses, tissue hypoxia/necrosis, and repair dysfunction. A critical common pathway is the persistent inflammatory response, which is characterized by neutrophil infiltration, pro-inflammatory cytokine storms (e.g., IL-6, TNF-α), and extracellular matrix destruction due to an imbalance between MMP-2/9 activity and collagen synthesis. These processes form a vicious cycle: the ulcer microenvironment promotes infection and further tissue breakdown, which in turn exacerbates inflammation and hypoxia, ultimately leading to progressive necrosis and the risk of non-healing ulcers or amputation. Created with WPS Word 2024. IL-6: Interleukin-6; MMP-2/9: matrix metalloproteinase-2/9; nIL-6: natural IL-6; TNF-α: tumor necrosis factor-alpha.

The pathological process of skin ulcer diseases is summarized in **Figure 1**.

## Mechanism of Ozone Therapy

### Direct oxidative damage and metabolic inhibition

Ozone mainly exerts broad-spectrum antibacterial effects through its powerful oxidative properties. Its mechanism includes direct structural damage and indirect metabolic inhibition. It oxidizes key metabolic enzymes such as DNA polymerase and respiratory chain enzymes, disrupting the cellular energy supply and biosynthesis processes and ultimately leading to bacterial death. Moreover, research has shown that ozone exposure significantly reduces the survival rates of methicillin-resistant *Staphylococcus aureus* and extensively drug-resistant *Pseudomonas aeruginosa*, and bacterial surface integrity is severely impaired.^15^

At the molecular level, the free radicals generated during ozone decomposition increase the level of intracellular reactive oxygen species, triggering oxidative stress. Ozone can interfere with the viability of most bacterial strains. Although it does not cause damage to bacterial membranes, an increase in reactive oxygen species levels can have adverse effects on lipid, protein, and DNA metabolism.^16^ It activates the nuclear factor erythroid 2-related factor 2/antioxidant response element pathway, upregulating key antioxidant enzymes, including superoxide dismutase (SOD) and glutathione peroxidase.^17^ An animal study revealed that ozone sunflower oil has a protective effect on ethanol-induced gastric ulcers in rats by stimulating several important antioxidant enzymes, such as SOD and glutathione peroxidase.^18^ However, its role in skin ulcers needs further research.

In addition to its antibacterial effect, ozone also destroys the structure of bacterial cell membranes (such as phospholipids and lipoproteins) by oxidizing them, thereby inhibiting growth and biofilm formation.^19^

### Enhanced biofilm penetration and clearance

Ozone nanobubble technology effectively overcomes the limitations of traditional ozone applications in multiple key aspects. First, by encapsulating ozone in hyaluronic acid-modified liposomes, the permeability and stability of ozone were significantly improved, enabling it to be more effectively targeted and delivered to deep tissues.^20^ Second, ozone can oxidize and decompose the extracellular polymeric substance matrix, thereby breaking down the physical barriers that biological membranes rely on for their existence. In addition, this technology can also interfere with the quorum sensing system of bacteria, suppress the generation of their aggregation signals, and promote the transition of biofilms from an attached state to a planktonic state.^21^

### Virus inactivation

Ozone can attack the capsid protein and nucleic acid chain of the virus and is effective against viruses; for example, treatment with approximately 1 ppm ozone (pH 7–6) can effectively reduce the viral load of feline calicivirus and Tulane virus, thereby reducing the recurrence rate.^22^

### Anti-inflammatory and immunomodulatory effects

Ozone can rebuild immune homeostasis at ulcer sites by regulating local oxidative stress levels and immune cell polarization, providing a basis for its potential to promote immune regulation and accelerate wound healing. Specifically, ozone treatment can increase the total number of white blood cells in ulcer sites and enhance the phagocytic function of granulocytes (such as neutrophils). In addition, ozone can activate macrophages and T lymphocytes while promoting the release of key cytokines such as interferon and ILs. These comprehensive effects collectively regulate the body’s immune system, enhance self-repair ability during wound healing, and lay the foundation for subsequent tissue repair.^23^ Ozone induces blood mononuclear cells to produce immunoregulatory cytokines, particularly granulocyte–macrophage colony-stimulating factor, IL-2, IL-8, interferon, and tumor necrosis factor-α.^24^ Ozone therapy enhances the expression of innate immune surface proteins such as CD14, CD11b, and Toll-like receptor 4; similarly, it also increases oxidative bursts and phagocytic function.^25^,^26^,^27^

### Immune cell polarization

Ozone treatment can induce macrophage polarization toward the M2 phenotype, promote IL-10 secretion, and inhibit the production of IL-6 and tumor necrosis factor-α.^28^ A review further confirmed that ozone intervention significantly increased the proportion of M2 macrophages in ulcerous tissues.^29^

### Angiogenesis and tissue repair

Ozone significantly promotes tissue regeneration,^24^ as it can drive the formation of new blood vessels, the reconstruction of the extracellular matrix, and the repair process of nerves. These positive changes are achieved via the activation of various signaling pathways related to tissue repair, including key pathways such as the vascular endothelial growth factor,^30^ cyclin D1,^31^ nuclear factor kappa-B,^26^ and nuclear factor erythroid 2-related factor 2/antioxidant response element^32^ signaling pathways. Ozone promotes tissue repair by increasing the expression of vascular endothelial growth factor and transforming growth factor-β and has anti-inflammatory properties.^33^,^34^ Ozone autohemotherapy can improve tissue hypoxia at the metabolic level by increasing the oxygen-carrying capacity of red blood cells and regulating the synthesis of nitric oxide.

### Hypoxia-inducible factor 1 alpha/vascular endothelial growth factor-A axis activation

Ozone can simulate hypoxic environments, thereby stabilizing the level of hypoxia-inducible factor-1 alpha, promoting the expression of vascular endothelial growth factor, and inducing the generation of capillaries. An *in vitro* experiment confirmed that, after ozone treatment, the mRNA level of hypoxia-inducible factor-1 alpha was increased compared with that in the control group, and the secretion of vascular endothelial growth factor was also increased.^35^

### Extracellular matrix remodeling

Ozone oil promotes fibroblast migration and epithelial mesenchymal transition by activating the PI3K/Akt/mTOR signaling pathway, thereby accelerating wound healing.^36^ The combination of ozone therapy and negative pressure wound treatment increases capillary density by accelerating protein and collagen synthesis after clearing dead bone while reducing bacterial colonization in the wound and promoting wound healing.^37^ The ability of ozone to promote ulcer wound repair is achieved through disinfection and sterilization, stimulating granulation tissue formation, accelerating angiogenesis, and detoxification.^38^ As an innovative technology, ozone has a positive effect on wound repair and nerve regeneration.^39^

### Stem cell recruitment and transduction

Ozone can significantly increase the migration speed of neural stem cells.^40^ Furthermore, ozone oxidation changes the stiffness of the extracellular matrix, activates the integrin-focal adhesion kinase signaling pathway, and thereby promotes cell migration and differentiation.

**Figure 2:**
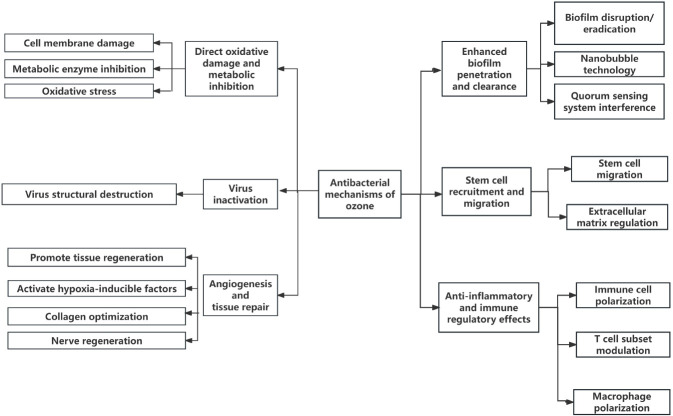
The multifaceted antibacterial mechanisms of ozone therapy. This study summarizes the key pathways through which ozone exerts its effects, including direct oxidative damage (e.g., cell membrane disruption, metabolic enzyme inhibition, and viral structural destruction), enhanced biofilm penetration via nanobubble technology and biofilm decomposition, immunomodulatory effects (such as macrophage polarization and T-cell subset modulation), and the promotion of tissue repair through angiogenesis, collagen optimization, nerve regeneration, and stem cell recruitment. Created with WPS Word 2024.

In summary, the mechanism of ozone therapy in the treatment of skin ulcerative diseases is multitargeted and interrelated (**Table 1** and **Figure 2**).

**Table 1:** Multitarget mechanisms of O_3_ therapy in skin ulcer healing

Mechanism	Key molecules/markers	Process	Effect/
Antibacterial	O_3_, reactive oxygen species	Directly oxidizes bacterial cell membranes and DNA; Stimulates local immune responses, enhancing phagocytosis	Inhibits bacterial growth, and reduces infection risk.
Anti-inflammatory	Pro-inflammatory cytokines, e.g., IL-1β, TNF-α Transcription factor, e.g., NF-κB	Inhibits release of IL-1β, and TNF-α Downregulates NF-κB signaling, reducing inflammatory signal transduction	Mitigates local inflammation, and reduces redness and pain
Immunomodulatory	Anti-inflammatory cytokines, e.g., IL-10, TGF-β Macrophages, lymphocytes	Promotes secretion of IL-10, and TGF-β Regulates immune cell activity, facilitating wound healing	Balances immune response, and prevents excessive tissue damage
Pro-angiogenic	Vascular endothelial growth factor, platelet-derived growth factor	Stimulates endothelial cell proliferation and migration, promoting neovascularization; Activates pro-angiogenic signaling pathways (e.g., PI3K/Akt)	Improves blood supply to the ulcer site, accelerates healing
Tissue repair	Fibroblasts, collagen	Enhances fibroblast proliferation and extracellular matrix synthesis; Activates TGF-β/Smad signaling for fibrotic repair	Strengthens tissue regeneration, and fills ulcer defects

IL: Interleukin; NF-κB: nuclear factor kappa-B; O_3_: ozone; PI3K/Akt: phosphatidylinositol 3 kinase/protein kinase B; TGF-β: tumor necrosis factor beta; TNF-α: tumor necrosis factor alpha.

## Application of Different Ozone Therapies in Skin Ulcer Treatment

### Local ozone application

#### Ozonated oil

Ozonated oil is formed by stabilizing and dissolving ozone in a vegetable oil carrier, such as olive oil or flaxseed oil, to create long-acting peroxides. This substance exerts continuous antibacterial and prorepair effects through mechanisms such as antibacterial and biofilm clearance, anti-inflammatory and immunomodulatory actions, and promoting angiogenesis and tissue repair.^41^ Compared with other drugs, its advantages include long-lasting release, high safety, convenient operation, and certain cost-effectiveness. A study has shown that ozonated oils can significantly promote granulation tissue formation in diabetic foot ulcers.^42^ However, the application of ozone oil also has certain limitations, including the following points: (1) Limited penetration depth: Ozone oil is less effective for treating deep tissue infections (such as osteomyelitis), and its efficacy in treating radiodermatitis ulcers is significantly lower than that of local ozone gas perfusion. (2) Stability depends on the carrier: There is a significant difference in the ozone loading capacity of different oils (olive oil > sunflower oil), requiring standardized preparation processes. (3) Not suitable for exudative wounds: The oil-based matrix may hinder the drainage of exudate, increasing the risk of infection. Clinical application suggestions are as follows: this technology is indicated for the treatment of superficial diabetic foot ulcers, pressure ulcers, and methicillin-resistant *Staphylococcus aureus*-infected wounds.^43^ The use of ozone oil with a concentration of 20–40 g/mL, which is applied topically once or twice a day, is recommended. Clinical practice has shown that combining the application of ozone oil with debridement treatment can further enhance the therapeutic effect. A new drug delivery system that forms a stable peroxide by dissolving ozone in medical oils such as olive oil or flaxseed oil has been successfully developed. The stability of this peroxide is attributed to lipid encapsulation technology, which effectively avoids the rapid decomposition of ozone.

#### Ozonated water

Ozonated water is a disinfectant formed by dissolving ozone in water. In the treatment of skin ulcers, ozonated water is mainly used for wound bed preparation and wound flushing. Its broad-spectrum antibacterial efficacy can effectively kill bacteria (including drug-resistant strains such as methicillin-resistant *Staphylococcus aureus*), viruses, fungi, and parasites, making it a unique advantage in treating wounds with severe contamination or severe biological colonization.^44^

#### Gaseous ozone

The mechanism of local ozone perfusion in the treatment of skin ulcer diseases involves multiple factors.^45^ First, ozone can react with polyunsaturated fatty acids in ulcer tissue to generate hydrogen peroxide and other lipid ozone products. These newly generated substances can activate the antioxidant enzyme system in the body and trigger a series of immune responses, which promote wound recovery. Second, the application of ozone can promote the synthesis of nitric oxide. Nitric oxide, as a vasodilator, can cause vasodilation, thereby improving blood perfusion in ulcer tissue and enhancing the delivery of oxygen and nutrients. In addition, ozone treatment can stimulate the body to produce various antioxidant enzymes, including SOD, glutathione peroxidase, glutathione S-transferase, and catalase. These enzymes play crucial roles in clearing harmful oxidants, reducing oxidative stress damage, and regulating inflammatory responses. Therefore, local ozone perfusion has multiple biological effects, including immune regulation, improved circulation, and antioxidant effects, making it a promising therapeutic approach.

A comparison of different local ozone applications in the treatment of skin ulcers is shown in **Table 2**.

**Table 2:** Comparison of different ozone treatments for skin ulcers

	Mechanism	Advantage	Limitation	Indication
Ozonated oils	Sustained oxidation, antibacterial, and repair promotion	Easy to use, high safety	Limited effect on deep infections	Diabetic foot ulcers, pressure ulcers
Ozone autohemotherapy	Systemic immune regulation, oxygen supply improvement	Synergy with systemic disease treatment	Requires professional equipment and operation	Complex ulcers with microcirculation disorders
Gaseous ozone perfusion	Deep tissue penetration	Rapidly kills anaerobic bacteria	Strict concentration control required	Necrotizing fasciitis with skin ulcers
Ozone bath	Surface cleaning and anti-inflammatory	Suitable for distal limb applications	Poor penetration, unstable efficacy	Superficial traumatic ulcers

### Systemic ozone application

Ozone autohemotherapy plays a role in the treatment of skin ulcers. The mechanism, advantages, and limitations of ozone autologous blood therapy for treating skin ulcers are summarized in **Table 3**.^46^,^47^,^48^,^49^,^50^

**Table 3:** Mechanisms, advantages and limitations of ozone autohemotherapy in the treatment of skin ulcers

Mechanism	(1) Immunomodulation and anti-inflammatory effects are achieved through macrophage polarization regulation and T-cell balance remodeling
	(2) Antioxidant stress and nuclear factor erythroid 2-related factor 2 pathway activation are achieved through the induction of endogenous antioxidant enzymes and the inhibition of nuclear factor kappa-β) inflammatory cascade
	(3) Improvement of tissue oxygenation and microcirculation.
	(4) Promotion of systemic metabolism and regeneration.
	(5) Synergistic anti-infection mechanisms. Strictly controlled ozone concentrations (20–40 μg/mL) trigger protective antioxidant responses, while concentrations greater than 60 μg/mL can lead to oxidative damage
Advantage	(1) Systemic effects: Addresses underlying pathologies (e.g., microcirculatory dysfunction in diabetes) not fully targeted by local therapies
	(2) Synergy with systemic conditions: Improves glycemic control in diabetic patients and reduces systemic inflammation
Limitation	Requires specialized ozone generators, sterile blood-handling systems, and trained personnel, which limits its accessibility.

### Photo-ozone combined therapy

Ozone combined with endovenous laser therapy utilizes the sensitization effect of light to enhance oxidative reactions in the body, while lasers can promote adenosine triphosphate synthesis in cells. Ozone gas combined with endovenous laser therapy showed improved efficacy for the treatment of lower limb venous ulcers and a lower recurrence ratio than endovenous laser therapy alone.^51^

**Figure 3:**
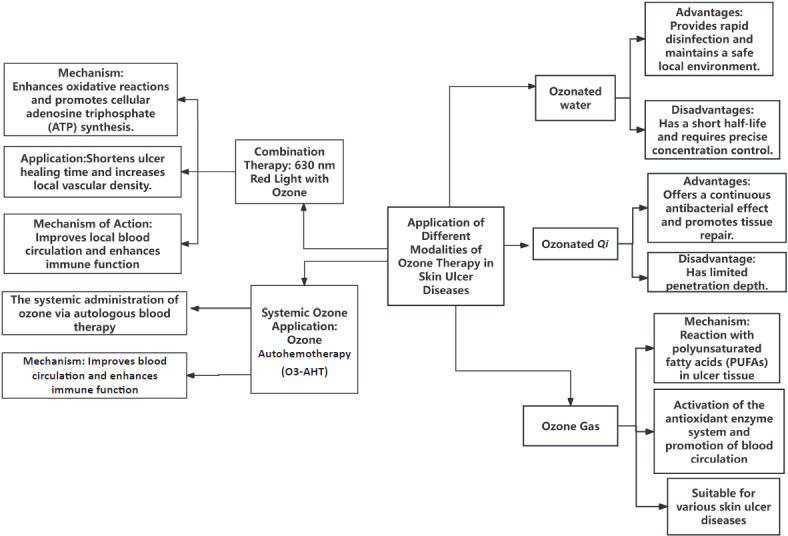
Characteristics of different forms of ozone in the treatment of skin ulcer diseases Various methods for the administration of ozone therapy, including local applications (ozonated oil and ozonated water), systemic ozone autohemotherapy, and a combined approach with 630 nm red light, are shown. Ozonated oil provides sustained antibacterial effects and promotes tissue repair but has limited penetration; ozonated water allows rapid disinfection but has a short half-life; autohemotherapy improves systemic circulation and immune function; and combination with red light enhances oxidative reactions and cellular energy (ATP) synthesis, accelerating ulcer healing. Collectively, these therapies act through mechanisms such as activating antioxidant enzymes and improving microcirculation to treat various skin ulcers. Created with WPS Word 2024.

In the treatment of cutaneous ulcer diseases, different forms of ozone therapy each have their own advantages and disadvantages (**Table 4** and **Figure 3**).

**Table 4:** Major adverse reactions to oxygen-ozone therapy in the treatment of skin ulcers

	Adverse reaction	High-risk factor	Prevention and control measure
Ozonated oil/water	Local tingling	Erythema damaged skin barrier	Wound debridement before use, concentration ≤ 40 μg/mL
Gaseous ozone	Perfusion periwound edema	Delayed Healing concentration > 60 μg/mL	Real-time concentration monitoring, single exposure < 15 min
Ozone autohemotherapy	Hemolysis dizziness	Glucose-6-phosphate dehydrogenase deficiency excessive concentration	Screen for contraindications before treatment concentration ≤ 40 μg/mL

## Clinical Evidence of Ozone Therapy in Different Skin Ulcerative Diseases

### Diabetic foot ulcers

Diabetic foot ulcers are a serious complication of diabetes, and their intractability is usually closely related to factors such as lower limb blood circulation disorders, neuropathy, and drug-resistant bacterial infections (such as methicillin-resistant *Staphylococcus aureus*).^52^ Research has shown that ozone therapy can promote the healing process of diabetic foot ulcers through three mechanisms.^53^ First, ozone can increase the local oxygen concentration in the wound, thereby improving microcirculation and accelerating the repair of damaged cells. Second, ozone can directly kill pathogenic microorganisms, especially those with significant inhibitory effects on drug-resistant bacteria, thereby effectively reducing the infection load of wounds. Finally, ozone also functions by regulating the levels of inflammatory factors, which helps to increase the regenerative ability of tissues. A study confirmed that ozone therapy can significantly reduce the amputation rate in the treatment of patients with diabetic foot infection.^54^ Another study has shown that the combined use of ozone therapy with conventional treatment can more effectively promote the complete healing of diabetic foot ulcers than can simple conventional treatment.^55^ Its mechanism mainly includes damaging bacterial cell membranes, inducing intracellular oxidative damage, and regulating the immune response. Ozone nanobubble therapy significantly improved the complete closure rate of wounds and provided sustained antibacterial effects with persistent stability and enhanced tissue permeability.^56^

### Chronic venous leg ulcers and refractory wounds

Existing evidence suggests that ozone therapy can promote the healing of venous ulcers by achieving several key therapeutic effects, including reducing the ulcer area, decreasing the bacterial load, and alleviating local inflammation.^57^ Local ozone therapy for venous leg ulcers accelerates the healing process of ulcers in objective planimetric assessments and reduces pain intensity.^58^ However, due to limitations in research quality and heterogeneity in treatment regimens (such as concentration, frequency and application methods), future randomized controlled trials with standardized parameters and strict designs are needed to ultimately determine their efficacy and safety.

In the field of burn wound management, the application of ozone effectively promotes wound healing by reducing tissue damage, increasing antioxidant enzyme activity, inhibiting infection, and promoting epithelial regeneration.^59^ A case report pointed out that the combination of ozone therapy and silver dressing can be used as an auxiliary means to treat refractory ulcers, especially diabetic foot ulcers.^60^ The synergistic effect of this combination therapy has significant potential for promoting wound healing and controlling infections.

### Other skin ulcer diseases

Ozone and its derivatives have shown multiple potential applications in the treatment of other refractory skin ulcers. Basic research has confirmed that plant oils treated with ozone, such as sunflower seed oil, have therapeutic effects on fungal ulcers. In addition, ozone therapy also has unique value in addressing severe skin complications caused by chemotherapy drugs such as doxorubicin.

## Differences Between Ozone Therapy and Other Therapies in Skin Ulcer Healing

A comparison of the advantages and disadvantages of ozone therapy and other treatment methods for the treatment of cutaneous ulcer diseases is shown in **Table 5.**

**Table 5:** Comparison of ozone and other therapies in the treatment of skin ulcers

	Mechanism	Advantage	Limitation
Oxygen-ozone therapy	Oxidative bactericidal, regulates oxygen metabolism	No drug resistance, dual effects of promoting repair and anti-infection	Requires strict concentration control
Antibiotics	Inhibits pathogen proliferation	Wide coverage of deep infections	Risk of drug resistance, no repair-promoting effect
Growth factor	Stimulates cell proliferation and migration	Precise targeting of repair signaling pathways	High cost, easy inactivation
Negative pressure wound therapy	Promotes wound healing	Closure rapid reduction of wound area	Limited efficacy on hypoxic ulcers

### Traditional antibiotic treatment

Although traditional antibiotic treatment can effectively inhibit bacterial proliferation, its clinical efficacy is often influenced by multiple factors. For example, the emergence of bacterial resistance (such as methicillin-resistant *Staphylococcus aureus*) and the lack of direct promotion of tissue repair ability by antibiotics themselves are the key reasons for their poor treatment effectiveness. In contrast, ozone can exert a broad-spectrum bactericidal effect by oxidizing the lipid components and enzyme system of bacterial cell membranes. A randomized controlled trial on methicillin-resistant *Staphylococcus aureus*-infected ulcers confirmed the effectiveness of ozone therapy. After 6 weeks of local ozone oil treatment, the ulcer healing rate of patients was as high as 82%, which was significantly better than that of the control group receiving vancomycin treatment (58%).^19^

Although ozone therapy has significant therapeutic effects, its ability to penetrate deep-tissue infections is usually weaker than that of systemic antibiotics. Therefore, to maximize the therapeutic effect, ozone therapy should be combined with systemic antibiotics to achieve synergistic effects.

### Bioactive dressings

Dressings containing growth factors such as platelet-derived growth factor and epidermal growth factor can promote accelerated formation of granulation tissue.^61^ However, this type of dressing has limitations such as high cost and poor stability. In contrast, ozone not only has tissue repair effects but also has anti-infective effects, which help promote the healing of skin ulcers.^62^ Research has shown that the combination of ozone and silver ions can reduce the number of methicillin-resistant *Staphylococcus aureus*^63^ and accelerate ulcer wound healing.^60^

### Physical therapy

Negative pressure wound therapy mainly promotes wound contraction through mechanical traction.^64^ However, this therapy has limitations in improving local microcirculation in wounds. In contrast, ozone autohemotherapy can improve tissue hypoxia at the metabolic level by increasing the oxygen-carrying capacity of red blood cells and regulating the synthesis of nitric oxide. Clinical research has shown that the combination of ozone autohemotherapy and negative pressure wound therapy for the treatment of venous ulcers can shorten the average healing time of patients, which is significantly better than that of patients treated with negative pressure wound therapy alone.^65^

## Safety and Risk Management of Ozone Therapy in Skin Ulcer Healing

### Safety and risks of local applications

The incidence of adverse reactions related to ozone therapy is relatively low, and the clinical manifestations of adverse reactions are mainly temporary local pain or erythema, which can usually be alleviated on their own within 24 hours. However, to achieve its clinical benefits, more high-quality research is still needed to further verify its safety, and standardization of treatment operation details is crucial to avoid potential medication risks. The ozone concentration is strictly limited (≤ 5% in gas mixtures, ≤ 100 mg/L in pharmaceutical oxygen carriers), and high concentrations of ozone (such as ozone in atmospheric pollution) are harmful to lung tissue.^66^

### Safety and risks of systemic treatment

The main potential risk of ozone autohemotherapy is a hemolytic reaction, which is directly related to high ozone concentrations (> 60 μg/mL) or improper mixing times of blood and ozone.^67^ Therefore, when ozone therapy is implemented, it is necessary to strictly control its concentration below 60 μg/mL. In addition, most treatment-related adverse events stem from local traumatic reactions caused by secondary infections or improper procedures. The contraindications for ozone therapy mainly include glucose-6-phosphate dehydrogenase deficiency (which poses a risk of inducing ozone-induced hemolytic crisis), hyperthyroidism (which may exacerbate oxidative stress), and pregnancy. The different forms of ozone therapy used to treat skin ulcer diseases and their main adverse reactions are summarized in **Table 6**. Overall, existing evidence suggests that ozone therapy for skin ulcer diseases is a relatively safe, effective, and cost-effective adjunctive treatment method under the premise of short-term and standardized application.^68^,^69^ However, it must also be acknowledged that high-quality safety data on the extremely long-term (several years or even longer) and high-frequency applications of ozone are still lacking. Most clinical studies have short follow-up times and have not fully achieved double-blind control. Whether the potential theoretical risks of ozone, such as genetic toxicity, will manifest in clinical treatment still requires more rigorously designed, long-term follow-up studies with large sample sizes. The concentration of ozone therapy should be selected based on the specific type and severity of the ulcer. For superficial ulcers, it is recommended to local ozone concentrations be controlled within the range of 20–40 μg/mL. When cases of deep infections such as cellulitis or osteomyelitis are encountered, the local ozone concentration can be increased to 60 μg/mL. Regardless of the local or systemic concentration, the ozone concentration needs to be controlled below 60 μg/mL. To alleviate the oxidative stress damage that may be caused by ozone therapy, the combined use of antioxidants is recommended. For example, research evidence suggests that pre-treatment with local vitamin E or C before ozone therapy can effectively reduce oxidative stress levels and may have a positive effect on ulcer healing.^70^

**Table 6:** Applications and adverse events of ozone therapy

	Application	Main adverse reactions
Ozone water therapy	Local flushing, wet compress or soaking of ulcer wounds, using the oxidative effect of ozone water to kill bacteria and promote wound healing.	1. Skin irritation (redness, swelling, pain); 2. Allergic reactions (itching, rash); 3. Excessive concentration may cause tissue damage.
Ozone oil therapy	Mixed ozone with vegetable oil to make ozone oil, apply it to ulcer wounds, form a protective barrier, and promote repair.	1. Local stimulation (burning sensation); 2. Allergic reactions (contact dermatitis); 3. Oil blockage may increase the risk of infection.
Ozone gas therapy	Generated ozone gas through an ozone generator, which directly acts on ulcer wounds or the surrounding environment, killing bacteria and improving the microenvironment.	1. Respiratory irritation (coughing, difficulty breathing); 2. Dizziness and headache; 3. High concentration exposure may lead to lung damage.
Ozone autohemotherapy	Extracted the patient’s blood, mixed with ozone, and then transfused back to regulate immunity and promote blood circulation through systemic effects.	1. Fever and chills; 2. Allergic reactions (which can lead to anaphylactic shock in severe cases); 3. Hemolysis reaction (destruction of red blood cells).

### Clinical challenges

The clinical application of ozone therapy for skin ulcers faces many challenges. First, due to the current lack of globally unified treatment guidelines, clinical doctors often rely more on personal experience rather than evidence-based medical standards when determining ozone concentration, treatment duration, and selecting combination therapy regimens. Second, there is a risk of gaseous ozone leakage during the treatment process, which not only pollutes the environment but also exposes medical personnel and patients to unnecessary exposure risks. In addition, accurately delivering ozone to deep tissues, such as complex ulcer lesions accompanied by bone infections, by existing equipment remains a technical challenge. Finally, long-term follow-up data to understand the long-term effects of ozone on the human body are lacking. However, in disease treatment research, the longest follow-up observation period for ozone is only 12 months in published clinical studies, so there is a lack of sufficient clinical data to rule out the potential impact of this risk on humans.

## Current Controversies and Future Perspectives on Ozone Therapy in Skin Ulcer Healing

Although ozone therapy has shown potential advantages in the treatment of skin ulcers, its clinical application still faces many controversies and challenges. Future multidisciplinary collaboration is needed to optimize technology and increase evidence.

### Future research directions

#### Construction of the precision treatment system

It is crucial to establish a refined dose model to improve the therapeutic accuracy of ozone oxygen therapy. The model should fully consider the differences in different disease types (such as diabetic foot ulcers and psoriasis ulcers), disease stages (acute and chronic) and local microbial loads. In addition, innovations in targeted delivery technology are key to improving the penetration of ozone into deep infection foci and enhancing the specificity of treatment. Specifically, the focus should be on developing new technologies, such as lipid-based sustained-release carriers, improving existing technologies (such as improving nanobubble ozone delivery systems), optimizing catalyst design (such as TiO_2_ modification) to increase system stability, exploring multiple technology combinations (such as photocatalysis combined with ozone microbubbles), etc. The goal of these technologies is to increase the permeability of ozone in deep infections such as osteomyelitis while minimizing potential systemic toxicity risks. Furthermore, exploring light-controlled ozone generation technology (such as near-infrared triggered release) is also highly important, as it is expected to achieve precise local treatment in time and space.

#### Multidisciplinary technology integration

In recent years, the application of ozone therapy has shifted from a single mode to a combined treatment strategy, aiming to overcome the bottleneck of the efficacy of single therapy through synergistic effects and provide a more optimized treatment scheme for refractory skin ulcers.

The combination of ozone therapy and conventional treatment methods has significant clinical potential. Low-intensity endovenous lasers activate the mitochondrial respiratory chain to improve cellular energy metabolism, whereas ozone improves the tissue oxygen supply by increasing oxygenation. The two complement each other in improving the tissue microenvironment and have been proven to synergistically accelerate the healing process of ulcers.^51^

The multi-target mechanism of ozone therapy combines anti-infection effects, the promotion of angiogenesis, and the regulation of the inflammatory response, making it uniquely advantageous in the treatment of refractory ulcers accompanied by drug-resistant bacterial infections or microcirculatory disorders. Compared with stem cell therapy, ozone therapy has better anti-infection effects and has a higher cost-effectiveness ratio.^71^ However, despite the broad prospects of combination therapy, its clinical application still faces challenges, and there is an urgent need to further standardize the treatment plan and systematically evaluate and verify its long-term safety.

Research has shown that there is a synergistic effect between ozone therapy and photodynamic therapy. An animal experiment confirmed that the combination of blue light and red light with ozone therapy can significantly accelerate the healing speed of methicillin-resistant *Staphylococcus aureus*-infected wounds.^72^

In addition, ozone therapy and stem cell therapy have shown potential for synergistic application. A study in rats explored the effects of ozone, chitosan hyaluronic acid, and mesenchymal stem cells on wound healing.^73^ The results showed that the combination of ozone, chitosan hyaluronic acid, and mesenchymal stem cells can effectively accelerate the healing process of rat wounds. However, although these preliminary results are encouraging, larger clinical trials are still needed to fully evaluate the effectiveness and safety of these synergistic factors to improve the treatment of chronic wounds.

The development of intelligent diagnosis and treatment systems is also highly important in the field of wound treatment. By integrating artificial intelligence wound imaging systems (such as 3D wound depth analysis technology), real-time feedback can be provided for the setting of ozone therapy parameters (such as concentration and exposure time), and dynamic adjustment can be supported to achieve more accurate and personalized treatment.

**Figure 4:**
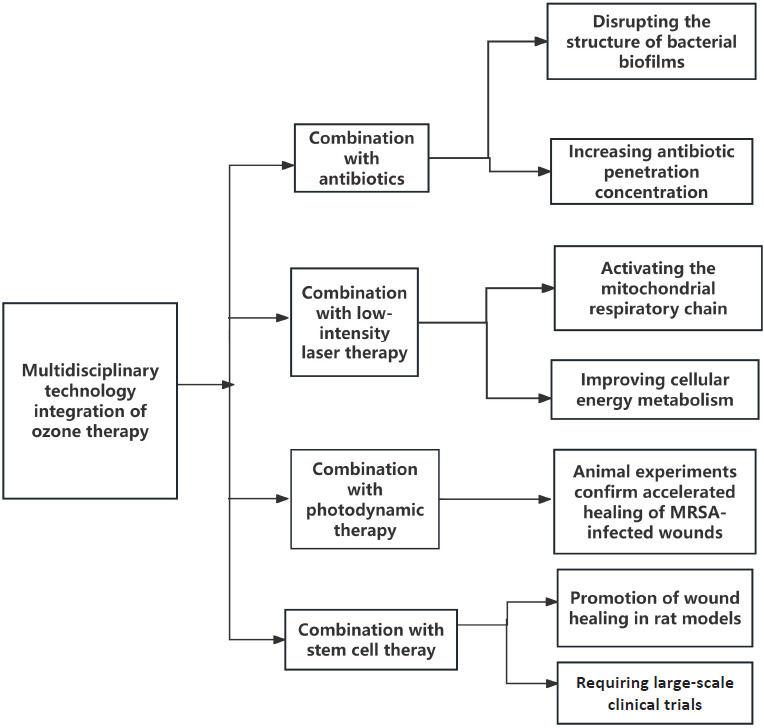
Multidisciplinary technology integration of ozone therapy for enhanced treatment of skin ulcers. The synergistic strategies of combining ozone therapy with other modalities enhance efficacy through multiple mechanisms: disrupting the bacterial biofilm structure, increasing antibiotic penetration, activating the mitochondrial respiratory chain to improve cellular energy metabolism, and accelerating wound healing. Animal studies have confirmed these benefits, showing improved healing in MRSA-infected wounds and diabetic rat models. Created with WPS Word 2024. MRSA: Methicillin-resistant *Staphylococcus aureus.*

The characteristics of the integration of ozone and other treatment methods are summarized in **Figure 4**.

#### Mechanism deepening and translational research

Future studies will use single-cell sequencing and spatial transcriptomics techniques to investigate the complex regulatory network of ozone on immune cells (such as macrophage polarization) and microbial communities (analyzed by 16S rRNA sequencing) in the ulcer microenvironment. To promote the clinical application of ozone combination therapy, high-throughput drug screening should be conducted based on existing research to optimize and establish efficient and reliable ozone combination therapy plans. At present, the transformation and application of ozone therapy in the field of skin ulcer treatment have entered a critical stage, and its development focus is transitioning from the early stage of “evidence accumulation” to the more important stage of “precision standardization.”

## Limitations

Although this systematic review comprehensively integrates and analyzes the existing evidence of ozone therapy in the management of skin ulcers, several inherent limitations in its design still need to be clarified. First, the literature search is limited to articles published in English, which may introduce language bias and result in the omission of potentially relevant research published in other languages. Second, although there is a systematic search strategy, excluding gray literature (such as ongoing clinical trials, conference abstracts, and unpublished data) and the inherent trend of negative results published in journals (publication bias) may bias the overall synthesis of evidence toward more favorable outcomes. In addition, the significant heterogeneity observed in the included studies, manifested in changes in ozone concentration (20–60 μg/mL), treatment methods (such as ozone oil and gas injection), treatment duration, and ulcer etiology (such as diabetes foot ulcers and venous leg ulcers), precluded formal meta-analysis. This heterogeneity limits the ability to draw clear and unified conclusions about the optimal treatment parameters. Finally, the author conducted quality assessment and data extraction. Although the program aims to minimize subjectivity, the lack of a broader, independent review panel may introduce bias when interpreting research results.

## Conclusion

Ozone therapy not only has significant antibacterial effects but also has a series of biological effects. It can stimulate the production of antioxidant enzymes such as SOD and catalase, increase the elasticity of red cell membranes, improve blood flow and tissue oxygenation, promote the release of growth factors, and activate cell metabolism and immune function. Through multi-target regulation, ozone therapy has shown significant efficacy in treating diseases such as diabetic foot ulcers and burns, with overall controllable safety. This study provides a new treatment strategy for ulcerative skin diseases. Although it has shown significant efficacy in the treatment of skin ulcer diseases such as diabetic foot ulcers and psoriasis, high-quality clinical studies are still needed to optimize treatment regimens and establish standardized application guidelines. In the future, combined with precision medicine and new delivery technologies, ozone is expected to play a more extensive role in the treatment of inflammatory diseases. Dose optimization, equipment standardization, and high-quality clinical evidence are still bottlenecks for its widespread application. Future research needs to focus on deepening the mechanism and standardizing the application to unleash the maximum therapeutic potential of ozone therapy in skin ulcer diseases.

## Data Availability

*Not applicable.*
